# Comparison of Monitored Anesthesia Care with Target-Controlled Infusion and Sevoflurane Mask Anesthesia for Outpatient Gynecologic Surgery: A Single-Center Prospective Randomized Controlled Study

**DOI:** 10.3390/medicina62030596

**Published:** 2026-03-21

**Authors:** Jaesuk Kim, Haneul Jeong, So Young Kwon

**Affiliations:** Department of Anesthesiology and Pain Medicine, St. Vincent’s Hospital, College of Medicine, The Catholic University of Korea, Seoul 16247, Republic of Korea

**Keywords:** anesthesia, inhalation, intravenous, gynecologic anesthesia, outpatient surgery, satisfaction, patient, respiratory depression, conscious sedation

## Abstract

*Background and Objectives*: Rapid recovery and patient comfort are key goals in ambulatory surgery. Although sevoflurane anesthesia is widely used, target-controlled infusion (TCI) with propofol and remifentanil has gained attention for its potential benefits. However, comparative data regarding recovery profiles and respiratory safety remain limited. *Materials and Methods*: In this prospective randomized controlled trial, 51 ASA I–II patients undergoing outpatient gynecologic surgery were assigned to either a TCI group (*n* = 25) or an inhalation mask (IM) group using sevoflurane and nitrous oxide (*n* = 26). Primary outcomes included time to postanesthesia care unit (PACU) discharge readiness and patient and surgeon satisfaction. Secondary outcomes included eye-opening time, anesthesia duration, PACU stay, and respiratory adverse events. *Results*: Time to Aldrete score ≥9 did not differ significantly between groups (*p* = 0.697). The IM group demonstrated faster eye opening (*p* = 0.002), while patient satisfaction was higher in the TCI group (*p* < 0.001). Surgeon satisfaction favored the IM group (*p* = 0.035). Respiratory depression occurred more frequently in the TCI group (28.0% vs. 0%, *p* = 0.012). *Conclusions*: Sevoflurane anesthesia allowed faster emergence, whereas TCI provided greater patient satisfaction but increased respiratory risk. Both techniques are feasible for ambulatory gynecologic surgery when appropriately selected. Trial registration: This study was retrospectively registered at the Clinical Research Information Service (CRIS), Republic of Korea (KCT0011352).

## 1. Introduction

Recent progress in surgical techniques has resulted in procedures that are increasingly less invasive, facilitating reduced hospital stays and expediting patient return to daily activities [1]. The rise in ambulatory and daycare surgery centers in recent years has further highlighted the importance of achieving rapid recovery with minimal complications [2,3,4]. From a cost-effectiveness standpoint, ensuring prompt recovery and minimizing anesthesia-related complications to enable patients’ rapid return to normal life has become increasingly significant [5].

In outpatient gynecologic surgery, inhalation anesthesia—specifically with sevoflurane—has traditionally been the standard approach due to its practicality and procedural familiarity [6]. However, to achieve more rapid and favorable recovery outcomes, intravenous anesthetics—particularly propofol—are being used with increasing frequency [7]. Nevertheless, it remains uncertain if substituting traditional inhalational anesthesia with propofol-based techniques significantly enhances patient outcomes in the context of day surgery [8].

The rapid expansion of ambulatory surgery has intensified the need for anesthetic techniques that balance procedural efficiency with patient comfort. While volatile anesthetics remain standard due to their predictable recovery profiles, the emergence of total intravenous anesthesia (TIVA) via target-controlled infusion (TCI) offers potential advantages in reducing postoperative side effects and enhancing the overall patient experience. However, the choice between these modalities often involves a trade-off between respiratory stability and sedation quality, necessitating a more granular comparison in specific surgical contexts such as minor gynecologic procedures.

An example of such an anesthesia delivery method is target-controlled infusion (TCI), which enables precise regulation of anesthetic depth through maintenance of consistent effect-site concentrations. TCI most often utilizes a propofol and remifentanil combination to provide both sedation and analgesia.

Propofol is extensively utilized for its rapid onset, reliable titration, and swift recovery profile, characteristics that are well-suited for ambulatory surgery. Nevertheless, because propofol does not have intrinsic analgesic properties, short-acting opioids are frequently added to address procedural pain.

Remifentanil, a synthetic μ-opioid receptor agonist, is highly suitable for this context because of its extremely short half-life, fast onset, and facile titration. Its pharmacokinetic properties allow for rapid adjustments and quick patient recovery, rendering it especially beneficial for brief procedures such as outpatient gynecologic surgeries. When used together, propofol and remifentanil facilitate controlled sedation and may reduce the risk of delayed awakening or lingering drug effects.

Consequently, this study aimed to compare TCI-based anesthesia to conventional sevoflurane-based inhalational anesthesia in patients undergoing outpatient gynecologic surgery. The primary focus was to assess differences in recovery time—specifically the time to achieve discharge readiness from the postanesthesia care unit—and the incidence of respiratory complications.

## 2. Methods

**Figure 1:**
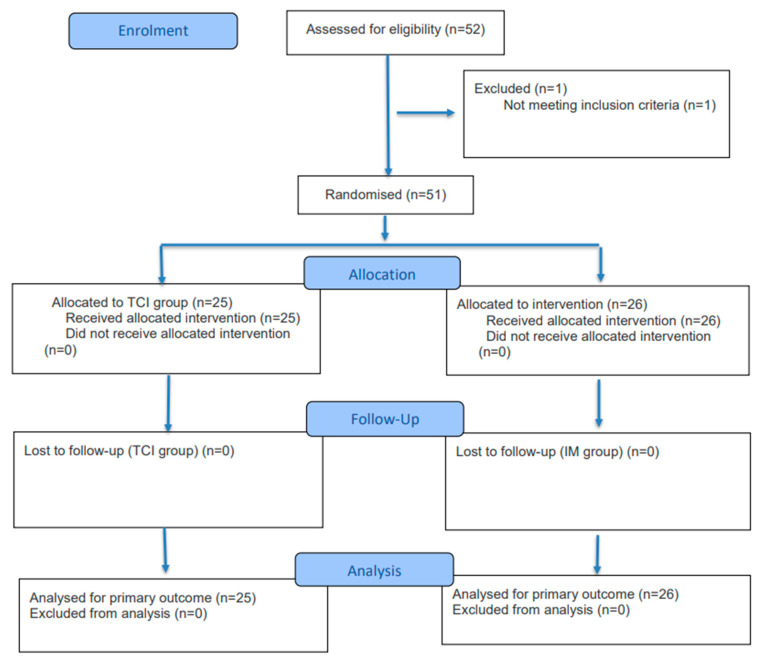
CONSORT 2025 flow diagram illustrating patient enrollment, randomization, allocation, follow-up, and analysis. A total of 52 patients were assessed for eligibility; one was excluded, and 51 were randomized into either the TCI group (*n* = 25) or the IM group (*n* = 26). All patients received the allocated intervention, completed follow-up, and were included in the final analysis.

This prospective, randomized controlled study was conducted at a single tertiary hospital after obtaining approval from the institutional review board (VC151MISI0152). The Ethics Committee approval date is 1 September 2015, with enrollment starting on 12 October 2015, and ending on 27 August 2019. The clinical trial was retrospectively registered at the Clinical Research Information Service (CRIS) of Korea. The registration number is KCT0011352. Regarding the clinical trial registration, this study was registered retrospectively. While the trial was conducted with strict adherence to the approved IRB protocol and ethical standards since 2015, the formal international registration was completed later to ensure the transparency and public accessibility of the data according to current journal policies. Written informed consent was acquired from all study participants. This study adheres to CONSORT reporting guidelines [9]. This study was conducted in accordance with the Declaration of Helsinki (2013 revision). A total of 52 patients with American Society of Anesthesiologists (ASA) Physical Status I or II, scheduled for elective gynecologic daycare procedures, were recruited. Patients were randomly allocated to the target-controlled infusion group (TCI, *n* = 25) or the inhalation mask group (IM, *n* = 26) by a sealed envelope technique. One patient was excluded after a revision in surgical plan necessitated a prolonged procedure, resulting in their omission from the final analysis. The flow of participants through the trial, including enrollment, randomization, allocation, follow-up, and analysis, is illustrated in Figure 1.

Patients were excluded if they declined or were unable to provide informed consent, had a prior history of sedative abuse, or had a known hypersensitivity to any of the administered anesthetic agents.

On entry to the operating room, standard monitors, including noninvasive blood pressure, heart rate, electrocardiogram (ECG), and peripheral oxygen saturation (SpO_2_), were applied. All patients maintained spontaneous ventilation throughout surgery.

TCI Group: Oxygen was administered at 5 L/min via a nonrebreather mask. Sedation commenced using a target-controlled infusion (TCI) pump (Orchestra, Fresenius Kabi, France) with propofol and remifentanil. The TCI pump was programmed using the Marsh model for propofol and the Minto model for remifentanil. The initial target effect-site concentration for propofol was 3 µg/mL, while remifentanil was set at 2 ng/mL. The depth of sedation was tracked by bispectral index (BIS) monitoring, with propofol titrated in 0.5 µg/mL increments to maintain BIS values within 40 to 60 (range: 1.5–4.0 µg/mL).

IM Group: Sedation in this group began with propofol (1 mg/kg), and anesthesia was maintained using sevoflurane in 50% nitrous oxide and oxygen, administered via mask ventilation, with adjustments made to maintain BIS values between 40 and 60. Sevoflurane was delivered via a calibrated vaporizer, starting at 2.0 vol% and titrated based on BIS values to ensure clinical stability.

The study’s primary endpoints were subjective satisfaction assessments collected from both patients and surgeons and the time taken to achieve discharge readiness from the PACU—defined as reaching an Aldrete score of ≥9.

Secondary outcomes comprised time to eye opening, total duration of anesthesia, length of surgery, PACU stay time, sedation quality measures, and rates of respiratory depression or other adverse events.

Respiratory depression was defined as an SpO_2_ value at or below 90% lasting more than 30 s. In cases where this occurred, interventions included titrating anesthetic dosages or providing assisted ventilation with a jaw thrust technique.

After completion of surgery, all anesthetic agents were discontinued. The time to eye opening was recorded beginning from the moment of verbal stimulation. Patients were subsequently transferred to the postanesthesia care unit (PACU), where monitoring was conducted using the modified Aldrete scoring system until they were deemed ready for discharge. The interval to achieve an Aldrete score ≥ 9 and the total duration of PACU stay were both documented. Upon discharge, patients evaluated their anesthetic experience using a 5-point scale, with 0 indicating extremely dissatisfied and 4 indicating extremely satisfied.

The depth of sedation during the procedure was also evaluated using a defined sedation score scale as follows:

(0) awake; (1) awake after verbal stimulation; (2) awake after light tactile stimulation; (3) awake after repeated shaking; (4) no response.

Furthermore, the operating surgeon assessed overall satisfaction with the surgical conditions using a 3-point scale:

(1) excellent condition for operation; (2) average condition for operation; (3) poor condition for operation.

### Statistical Analysis

The sample size was determined based on the primary outcome of patient satisfaction. According to a previous study on monitored anesthesia care for hysteroscopy, the standard deviation for patient satisfaction scores was approximately 0.8 on a 5-point scale [10]. To detect a 1-point difference in the satisfaction score between the TCI and IM groups with a significance level (α) of 0.05 and a power (1 − β) of 0.80, a minimum of 20 patients per group was required. Considering a potential dropout rate of 30%, we recruited 26 patients for each group, resulting in a total target sample size of 52 participants. A conservative dropout rate of 30% was prospectively chosen to account for the high unpredictability of ambulatory gynecologic surgery, such as sudden procedure cancelations or unexpected conversions to more invasive techniques (e.g., laparoscopy) that would necessitate different anesthetic management.

All statistical analyses were conducted with SPSS version 18.0 (SPSS Inc., Chicago, IL, USA). Normality of data distribution was assessed using the Shapiro–Wilk test. For continuous variables—including age, surgery duration, time to eye opening, time to Aldrete score ≥9, PACU stay, and satisfaction scores—comparisons between groups were performed using independent sample t-tests. Categorical variables, including ASA classification, incidence of respiratory depression, and occurrence of adverse effects, were assessed with the chi-square test. Statistical significance was defined as a *p*-value less than 0.05.

## 3. Results

After the exclusion of one patient due to a change in the surgical procedure, 51 patients remained in the final analysis. These patients were randomized into two groups: 26 in the inhalation mask (IM) group and 25 in the target-controlled infusion (TCI) group.

Baseline demographic characteristics such as age, height, and weight were comparable between both groups. The ASA physical status classification indicated a higher proportion of ASA II patients in the TCI group compared to the IM group; however, this difference did not reach statistical significance (*p* = 0.068, chi-square test). Table 1 provides a summary of the baseline characteristics.

### 3.1. Recovery-Related Parameters

Regarding recovery profiles, the IM group demonstrated a significantly shorter time to eye opening (5.71 ± 1.69 min) compared to the TCI group (7.93 ± 2.92 min, *p* = 0.002), indicating faster early emergence. However, this initial difference did not translate into faster discharge readiness from the PACU. The time to achieve an Aldrete score ≥9 was 8.56 ± 4.18 min in the IM group and 9.00 ± 3.75 min in the TCI group, showing no statistically significant difference between the two techniques (*p* = 0.697). Similarly, total anesthesia duration (*p* = 0.157), surgery duration (*p* = 0.359), and the overall length of stay in the PACU (*p* = 0.860) were comparable, with no significant clinical or statistical variations observed between the groups. A summary of these data is shown in Table 2.

### 3.2. Patient and Surgeon Satisfaction

In terms of subjective assessments, the TCI group reported markedly higher patient satisfaction scores (3.68 ± 0.56) than the IM group (2.31 ± 0.88, *p* < 0.001), suggesting a superior patient experience with intravenous sedation. Conversely, surgeon satisfaction was significantly better in the IM group (1.77 ± 0.59 vs. 1.32 ± 0.85, *p* = 0.035), likely due to the more stable surgical conditions and lack of respiratory interruptions. Notably, while the sedation quality score was numerically higher in the TCI group (3.12 ± 0.97) compared to the IM group (2.46 ± 1.39), this trend did not reach statistical significance (*p* = 0.056), indicating that both methods provided clinically acceptable sedation depth. Table 3 summarizes these findings.

### 3.3. Adverse Events

Safety analysis revealed that respiratory depression occurred in 7 patients (28.0%) in the TCI group, whereas no such events were recorded in the IM group (*p* = 0.012), highlighting the need for vigilant airway monitoring when using remifentanil–propofol TCI. For other postoperative complications, the incidence of nausea and agitation was low in both groups (7.7% in IM and 12.0% in TCI). The statistical analysis confirmed no significant difference in the occurrence of these minor adverse events (*p* = 0.220), suggesting that neither technique posed a significantly higher risk for common postoperative discomforts. Table 4 provides a summary of adverse event data.

## 4. Discussion

This prospective randomized trial was designed to evaluate and compare the anesthetic characteristics of target-controlled infusion (TCI) using propofol and remifentanil versus conventional inhalational mask (IM) anesthesia with sevoflurane in ambulatory gynecologic surgery. The primary outcome measured was the time required to achieve readiness for PACU discharge (Aldrete score ≥9). Secondary outcomes included time to emergence, satisfaction assessments, sedation quality, and incidence of adverse respiratory events.

The TCI group experienced a slightly longer time to achieve Aldrete score ≥9, but the difference was not statistically significant (*p* = 0.697). The IM group demonstrated significantly faster emergence, whereas the TCI group exhibited higher patient satisfaction but also a greater frequency of respiratory depression. These results suggest an interplay between clinical efficiency and subjective patient perception.

The significantly shorter time to eye-opening observed in the IM group corroborates findings from prior research, which indicate that volatile anesthetics like sevoflurane are associated with rapid early recovery owing to their eliminative properties via the lungs [11,12]. Conversely, TCI with propofol—particularly when administered with remifentanil—may result in slightly delayed emergence.

This observation is in agreement with Daccache et al. (2025), who, in their meta-analysis, noted that although total intravenous anesthesia (TIVA) utilizing TCI confers certain advantages in sedation management, inhalational anesthesia (IA) typically allows swifter recovery and lower costs, especially for brief ambulatory operations [13].

Interestingly, patient satisfaction was significantly higher in the TCI group (3.68 ± 0.56 vs. 2.31 ± 0.88, *p* < 0.001) despite slower emergence. This may be attributed to a smoother sedation course, lower frequency of intraoperative awareness, or less postoperative agitation—benefits frequently linked to propofol-based anesthesia [13]. The sedation quality score also demonstrated a trend toward higher values in the TCI group, which further supports the hypothesis that controlled intravenous sedation can improve perceived patient comfort.

In contrast, surgeon satisfaction was higher in the IM group (1.77 ± 0.59 vs. 1.32 ± 0.85, *p* = 0.035), possibly reflecting greater consistency in anesthetic depth and a reduced incidence of intraoperative respiratory complications. This presents a clinical challenge: while TCI may favor patient comfort, it could impede certain procedure-related requirements without vigilant management of respiratory variables.

The discrepancy between patient and surgeon satisfaction highlights a key trade-off. While patients preferred the smooth induction and lack of pungent gas odor associated with TCI, surgeons favored the IM group’s respiratory stability, which allowed for a more focused surgical field without the interruption of managing transient desaturations.

A notable and clinically relevant observation was the substantially increased incidence of respiratory depression in the TCI group (28.0% vs. 0%). This outcome is consistent with established risks associated with remifentanil, a potent μ-opioid receptor agonist, particularly in the setting of spontaneous ventilation without airway protection [14]. In our study, although respiratory depression occurred in 28.0% of the TCI group, all episodes were transient and effectively managed with simple maneuvers such as jaw thrust or minor dose titration, without any long-term sequelae. While remifentanil’s short duration and reversibility mitigate long-term consequences, even transient desaturation may prompt airway intervention, underscoring the necessity for close monitoring during TCI use in ambulatory cases.

This safety issue is further supported by Daccache et al.’s review, which stressed the importance of rigorous respiratory monitoring in TCI-based TIVA, specifically for patients not undergoing intubation [13]. Regarding the choice of airway management, although laryngeal mask airways (LMAs) could provide a more secure airway, we prioritized the non-invasive nature of mask anesthesia and spontaneous ventilation to facilitate faster discharge and minimize pharyngeal discomfort in this brief daycare procedure (mean duration < 9 min). Furthermore, our findings are consistent with previous literature where remifentanil–propofol combinations resulted in respiratory depression rates of up to 33% during hysteroscopy [10].

There were no significant group differences in anesthesia time, procedure duration, or PACU length of stay, reinforcing the practicality of both approaches in outpatient gynecologic procedures.

Our results indicate that both anesthetic strategies are practical for outpatient gynecologic surgery, though each offers distinct advantages: IM anesthesia may be optimal for high-throughput settings with minimal airway management, whereas TCI appears more favorable for settings in which patient comfort and reduced agitation are critical.

Of note, Daccache et al. recommend distinguishing between manual TIVA and TCI-based TIVA, as differences in recovery kinetics and complication profiles can be clinically significant [13]. As our investigation exclusively addressed TCI, it does not inform this distinction, pointing to the need for future comparative studies.

Several limitations should be acknowledged for this study. The sample size was relatively small. The 30% dropout rate used for sample size calculation might be higher than in other surgical settings; however, it was necessary to ensure sufficient statistical power given the nature of day-surgery protocols, such as unexpected cancelations or procedural changes. Additionally, randomization was not performed with stratification by ASA class, raising concerns about possible selection bias. The use of continuous analysis for satisfaction scores, despite their ordinal basis, may also affect interpretation. Additionally, the absence of a manual TIVA comparator constrains the generalizability of these findings across all intravenous anesthesia techniques.

Future studies should directly compare TCI and manual infusion, assess long-term outcomes such as PONV and cognitive function, and stratify participants according to ASA classification to account for baseline comorbidity differences.

## 5. Conclusions

Compared to inhalational anesthesia with sevoflurane, which facilitated more rapid early emergence, TCI with propofol and remifentanil yielded higher patient satisfaction despite an increased incidence of respiratory depression. These results indicate that the selection of anesthetic in ambulatory gynecologic surgery should reflect institutional priorities: sevoflurane may be preferred for rapid patient turnover, while TCI may be chosen for improved patient comfort, provided that robust monitoring for respiratory compromise is maintained.

## Figures and Tables

**Table 1:** Demographic Characteristics of the Two Groups.

	Group M	Group T	*p* Value
Patients (n)	26	25	
Age (years)	50.96 ± 9.10	51.84 ± 12.05	0.771
Height (cm)	158.31 ± 5.73	155.90 ± 6.31	0.161
Body weight (kg)	58.21 ± 9.17	55.96 ± 8.42	0.358
ASA PS class (I/II)	19/7	11/14	0.068

Data are presented as mean ± standard deviation or number of patients.

**Table 2:** Recovery and Procedural Times in IM vs. TCI Groups.

	Group M	Group T	*p* Value
Time to Aldrete ≥9 (min)	8.56 ± 4.18	9.00 ± 3.75	0.697
Time to eye opening (min)	5.71 ± 1.69	7.93 ± 2.92	0.002 *
Duration of anesthesia (min)	21.46 ± 5.16	23.76 ± 6.19	0.157
Time in PACU (min)	31.00 ± 6.27	30.72 ± 4.95	0.860
Duration of surgery (min)	8.19 ± 3.12	7.52 ± 1.94	0.359

Data are presented as mean ± standard deviation. * *p* < 0.05.

**Table 3:** Satisfaction and Sedation Quality Scores.

	Group M	Group T	*p* Value
Patient satisfaction score (0–4)	2.31 ± 0.88	3.68 ± 0.56	<0.001 *
Surgeon satisfaction score (0–2)	1.77 ± 0.59	1.32 ± 0.85	0.035 *
Sedation quality score (0–4)	2.46 ± 1.39	3.12 ± 0.97	0.056

Data are presented as mean ± standard deviation. * *p* < 0.05.

**Table 4:** Adverse Events in the Two Groups.

	Group M (*n* = 26)	Group T (*n* = 25)	*p* Value
Incidence of respiratory depression	0 (0%)	7 (28.0%)	0.012 *
Other adverse events (nausea, agitation)	2 (7.7%)	3 (12.0%)	0.220

Data are presented as number of patients (%). * *p* < 0.05.

## Data Availability

The original contributions presented in this study are included in the article. Further inquiries can be directed to the corresponding author.
